# The phylogenomic landscape of extended-spectrum β-lactamase producing *Citrobacter* species isolated from surface water

**DOI:** 10.1186/s12864-023-09867-4

**Published:** 2023-12-07

**Authors:** Lee-Hendra Chenhaka, Deidré A.B. Van Wyk, Charlotte Mienie, Cornelius C. Bezuidenhout, Kgaugelo E. Lekota

**Affiliations:** 1 Unit for Environment Science and Management, Microbiology, North-West University, Potchefstroom campus, Private Bag X6001, Potchefstroom, 2520 South Africa; 2 Unit for Environment Science and Management, Microbiology, North-West University, Mahikeng campus, Private Bag X2046, Mahikeng, 2745 South Africa

**Keywords:** ESBL-producing *Citrobacter*, Whole-genome sequencing, Antibiotic resistance, Surface water

## Abstract

**Background:**

*Citrobacter* species are Gram-negative opportunistic pathogens commonly reported in nosocomial-acquired infections. This study characterised four *Citrobacter species* that were isolated from surface water in the North West Province, South Africa.

**Results:**

Phenotypic antimicrobial susceptibility profiles of the isolates demonstrated their ability to produce the extended-spectrum β-lactamase (ESBL). Whole genomes were sequenced to profile antibiotic resistance and virulence genes, as well as mobile genetic elements. *In silico* taxonomic identification was conducted by using multi-locus sequence typing and average nucleotide identity. A pangenome was used to determine the phylogenomic landscape of the *Citrobacter* species by using 109 publicly available genomes. The strains S21 and S23 were identified as *C. braakii*, while strains S24 and S25 were *C. murliniae* and *C. portucalensis*, respectively. Comparative genomics and sequenced genomes of the ESBL-producing isolates consisted of n = 91; 83% *Citrobacter* species in which *bla-*_*CMY−101*_ (n = 19; 32,2%) and *bla-*_*CMY−59*_ (n = 12; 38,7%) were prevalent in *C. braakii*, and *C. portucalensis* strains, respectively. Macrolide (*acrAB*-TolC, and *mdtG*) and aminoglycoside (a*crD*) efflux pumps genes were identified in the four sequenced *Citrobacte*r spp. isolates. The quinolone resistance gene, *qnrB13*, was exclusive to the *C*. *portucalensis* S25 strain. *In silico* analysis detected plasmid replicon types IncHI1A, IncP, and Col(VCM04) in *C. murliniae* S24 and *C. portucalensis* S25, respectively. These potentially facilitate the T4SS secretion system in *Citrobacter* species. In this study, the *C. braakii* genomes could be distinguished from *C*. *murliniae* and *C. portucalensis* on the basis of gene encoding for cell surface localisation of the CPS (*vexC*) and identification of genes involved in capsule polymer synthesis (*tviB* and *tviE*). A cluster for the salmochelin siderophore system (*iro-BCDEN*) was found in *C. murliniae* S24. This is important when it comes to the pathogenicity pathway that confers an advantage in colonisation.

**Conclusions:**

The emerging and genomic landscapes of these ESBL-producing *Citrobacter* species are of significant concern due to their dissemination potential in freshwater systems. The presence of these ESBL and multidrug-resistant (MDR) pathogens in aquatic environments is of One Health importance, since they potentially impact the clinical domain, that is, in terms of human health and the agricultural domain, that is, in terms of animal health and food production as well as the environmental domain.

**Supplementary Information:**

The online version contains supplementary material available at 10.1186/s12864-023-09867-4.

## Background

*Citrobacter* species are characterised as Gram-negative, facultative anaerobe non-spore-forming rods that utilise citrate as the sole carbon source, and they belong to the family *Enterobacteriaceae* [1]. They are present in many environments, including water, soil, agriculture and food [2]. However, most species have been isolated from urinary and respiratory tract infections [3]. These *Citrobacte*r species have also shown resistance to the common antimicrobials that are used to treat individuals who suffer from these infections [4].

Moreover, *Citrobacter* species can colonise the intestinal tract of humans and animals [5, 6], as they consist of virulence factors such as the Vi capsule polysaccharide of *Salmonella enterica* subsp. *enterica* serovar Typhi str. In its turn, CT18 (*Salmonella Typhi*, for short) plays a role in pathogenicity. *Citrobacter* species can be identified in terms of different metabolic and virulence gene associations. These associations are evidenced by species such as *C. korseri*, where macromolecular secretion systems and pathogenic islands occur [4]. The pathogenic islands involved in the salmochelin siderophore system (*iroBCDEN* gene cluster) for colonisation can also be acquired by means of horizontal gene transfer in some members of the family of *Enterobacteriaceae* [7]. These genes confer a competitive advantage for colonisation during infections [8].

Based on microbiological phenotypic methods and DNA hybridisation technology, the genus *Citrobacter* has been classified into 11 species [9]. The genus was further expanded to a total of 16 species based on whole-genome sequencing technology [10, 11]. Most of the phenotypical features of *Citrobacter* species mimic *Escherichia coli*, so that they are readily misidentified when phenotypic microbiological tests are used [3, 12]. The use of 16 S ribosomal RNA (rRNA) sequences and multi-locus sequence analysis (MLSA) can discriminate among the genera of *Enterobacteriaceae* [13, 14]. However, the use of 16 S rRNA gene sequencing does not enable differentiation among species [15–18]. Therefore, whole-genome sequencing has become the preferred genotypic tool for surveillance and detection of possible outbreaks of infections by *Citrobacter* species in the ecosystem [19–22]. This tool provides a higher resolution of phylogenetic relationships, as it can trace common infections from a One Health perspective [23, 24].

*Citrobacter* species that are multi-drug resistant, namely *C. freundii*, *C. koseri*, *C. braakii* and *C. youngae*, were recently identified and reported in the Democratic Republic of the Congo, Benin, Nigeria, Algeria, Tunisia and South Africa [25]. In South Africa, particularly in the Mooi River system of the North West Province, studies have shown the presence of multi-drug resistant bacteria [26–28]. The system flows through a combination of rural and urban residential settlements and is mainly influenced by urban, mine and agricultural activities. The latter two drivers contribute 5% towards the global domestic product (GDP) of South Africa [29]. Livestock farming is believed to play a part in the distribution of *Citrobacter* spp. in river systems, possibly by means of faecal run-off [30, 31]. The health problems caused by waterborne pathogen bacteria belonging to the *Enterobacteriaceae* are aggravated by the rise of the antimicrobial-resistant bacteria phenomenon. This phenomenon has been identified as one of the most considerable health challenges globally [32–34].

Many *Enterobacteriaceae* have been characterised as displaying the ability to produce ESBL [35]. These ESBLs are enzymes that can inactivate and hydrolyse clinically relevant third generation antibiotics such as cephalosporins and penicillins [36]. A whole-genome sequencing approach facilitates genomic profiling of ARBs and a comprehensive investigation of their shared genes. The genetic diversity of *Citrobacter* species strains can also be inferred by means of this approach. Environmental samples are often overlooked and could be the main drivers for AR that can be transmitted to animals and humans [37–39]. The present study characterised ESBL-producing *Citrobacter* species obtained from surface river water in the Mooi River, North West Province, by using antibiotic susceptibility testing, screening for ESBL production and whole-genome sequencing.

## Results

### Microbiological tests and antimicrobial resistance profiles

Based on their inability to ferment sorbitol, the four isolates were presumptively identified as pathogenic *E. coli* species on selective differential SMAC-CT agar. All isolates were characterised as ESBL-producing species upon observing sub-culturing on CHROMagar ESBL media. The metallic blue colonies with reddish halo formation indicated the presence of *Klebsiella*, *Enterobacter* or *Citrobacter* species. Furthermore, the Enteropluri results showed similar biochemical reactions between isolates S21 and S24. S25 was the only isolate that was able to produce hydrogen sulphide. All four isolates were able to utilise citrate as a carbon source. On the basis of a disc diffusion assay (DDA), the antimicrobial patterns reflected in Table 1 showed isolates’ resistance to ampicillin, amoxicillin and erythromycin, except for *C. murliniae* strain 24. All the isolates examined in the present project, apart from *C. murliniae*, were classified as multi-drug resistant (MDR), since they were resistant to beta-lactams, macrolides and aminoglycosides.

**Table 1:** Phenotypic antimicrobial susceptibility profile of the four ESBL-producing *Citrobacter* species

Species ID	ESBL	Antibiotic Susceptibility Profile											
		β-lactams			Aminoglycosides				Tetracycline	Macrolide	Phenicol	Quinolone	
		AMX^3^	AMP^3^	CFZ^1^	KAN^1^	NEO^1^	STR^1^	GEN^2^	TET^1^	ERY^1^	CHL	NAL^1^	CIP^2^
*C. braakii* S21	+	R	R	R	I	I	R	S	I	R	S	S	S
*C. braakii* S23	+	R	R	R	R	R	R	S	S	R	S	I	S
*C. murliniae* S24	+	I	I	S	S	S	S	S	S	R	S	S	S
*C. portucalensis* S25	+	R	R	R	I	S	I	S	S	R	S	R	S

Table 1 Definitions: KAN: kanamycin; NEO: neomycin TET: tetracycline; ERY: erythromycin; GEN: gentamycin; CIP: ciprofloxacin; AMX: amoxicillin; CFZ: cefazolin; CHL: chloramphenicol; STR: streptomycin; NAL: nalidixic; AMP: ampicillin (https://journals.asm.org/abbreviations-conventions, accessed on 1 March 2023)

Definitions: 1 = 1st generation; 2 = 2nd generation; 3 = 3rd generation; R = resistant; I = intermediate; S = susceptible

### Genomic features and whole genome in silico taxonomic analysis

The scaffolds engendered by the sequenced *Citrobacter* genomes showed between 62 and 144 contigs (Table 2). Furthermore, pubMLST showed that genome strains S21 and S23 were *C. braakii*, while strains S24 and S25 were *C. murliniae* and *C. portucalensis*, respectively. The use of ANI showed that S21 and S23 were *C. braakii* (ANI > 98%). Strain S24 had a 99.26% ANI when the *C. murliniae* strain P080C CL was used as a reference. Strain S25 was identified by means of pubMLST as *C. portucalensis* and was found to share 90% of its identity with most of the *C. freundii* genomes. ANI classified this strain as *C. portucalensis* when *C. portucalensis* A60^T^ was used as a reference.

**Table 2:** Genome features of the four sequenced *Citrobacter* species

Strain	Assembly size (bp)	Contigs	G + C content	N50	CDS	ANI (%)	PubMLST species ID	ARGs No.	VGs No.	Insertion Sequence	Trans-poson	T4CP gene cluster	T4SS secretion system	Plasmid ID and [Type]
**S21**	4 987 947	144	52.12	60,775	4923	*C. braakii* (98.6) *	*C. braakii*	25	56	*IS5* *IS102* *IS5075*	Tn5403	-	*VirB1, VirB2*, *VirB3, VirB4*, *VirB5, VirB6*, *VirB8, VirB9*, *VirB10, VirB11*, *VirD4*	AA885 [rep_1804]
**S23**	4 805 424	62	52.17	145,585	4602	*C. braakii* (98.7) *	*C. braakii*	24	55	*-*	-	Present	*-*	-
**S24**	5 481 339	104	50.62	192,439	5492	*C. murliniae* (99.26) **	*C. murliniae*	24	47	*ISEsa1* *ISEsa2* *ISKpn24* *ISKpn26* *IS5075* *ISEc33*	Tn5403	Present	*VirD4-TraABCD* *TraEFGH-TrbABCD* *TrbEFGH* *TrbIJKLV%* *TraX_F-TrbBCDE* *TrbFGHIJLN%%*	AA543 [IncHI1A, IncP] AA810 [rep_cluster_1368] AC978 [-] AC431 [-]
**S25**	5 269 614	91	51.49	154,085	5352	*C. portucalensis* (98.6) ***	*C. portucalensis*	24	38	*ISSpu2* *ISEsa1* *ISEsa2* *IS903* *IS5075* *ISKox1*	Tn5403	Present	*TraABCEFG* *TraHIKLNVW#*	AB130 [Col(VCM04)] AA423 [-] AF384 [-] AF578 [-]

*Citrobacter braakii strain DY2019 was used as a reference; ** *Citrobacter murliniae* strain P080C CL was used as a reference;*** *Citrobacter portucalensis* A60T was used as a reference; ARG: antibiotic resistance genes, VGs: virulence genes; IS: insertion sequence (isfinder); Tn: transposons (tn_registry), (-) indicates absent; % Type IV secretion system gene cluster determined in plasmid AA543; %% Type IV secretion system gene cluster determined in plasmid AC978; # Type IV secretion system gene cluster determined in plasmids AB130; No. is the number of genes; CDS: coding domain sequences; C: contigs

The sequenced *C. braakii* strains S21 and S23 have genomes that are 4.98 Mb and 4.80 Mb in size, respectively. Different plasmids replicon types were present in the *C. murliniae* strain S24 and *C. portucalensis* strain S25. These genomes were larger (> 5.2 Mb) than the *C. braakii* genomes sequenced in this study. This was confirmed by the high number of coding sequences (CDS) in these two strains. The GC content of *Citrobacter* genomes ranged between 50% and 52%. The GC content of sequenced *C. braakii* genomes was approximately 52%, which was the same as the reference strain *C. braakii* ATCC 8090^T^ and *C. braakii* strain DY2019 of the present study. The GC of the *C. murliniae strain* S24 was 50.62%, which was the same as *the C. murliniae* strain PC080C CL as isolated from human faeces available in GenBank. However, the genome size of *C. murliniae* strain PC080C CL was 5.0 Mb, which was slightly lower than that of *C. murliniae* strain S24 as sequenced in the present study. The genome size of the *C. portucalensis* strain S25 was 51.49%, which was similar to the *C. portucalensis* A60^T^ reference strain.

### Pangenomics and placement of the four sequenced *Citrobacter* species isolates

**Fig. 1:**
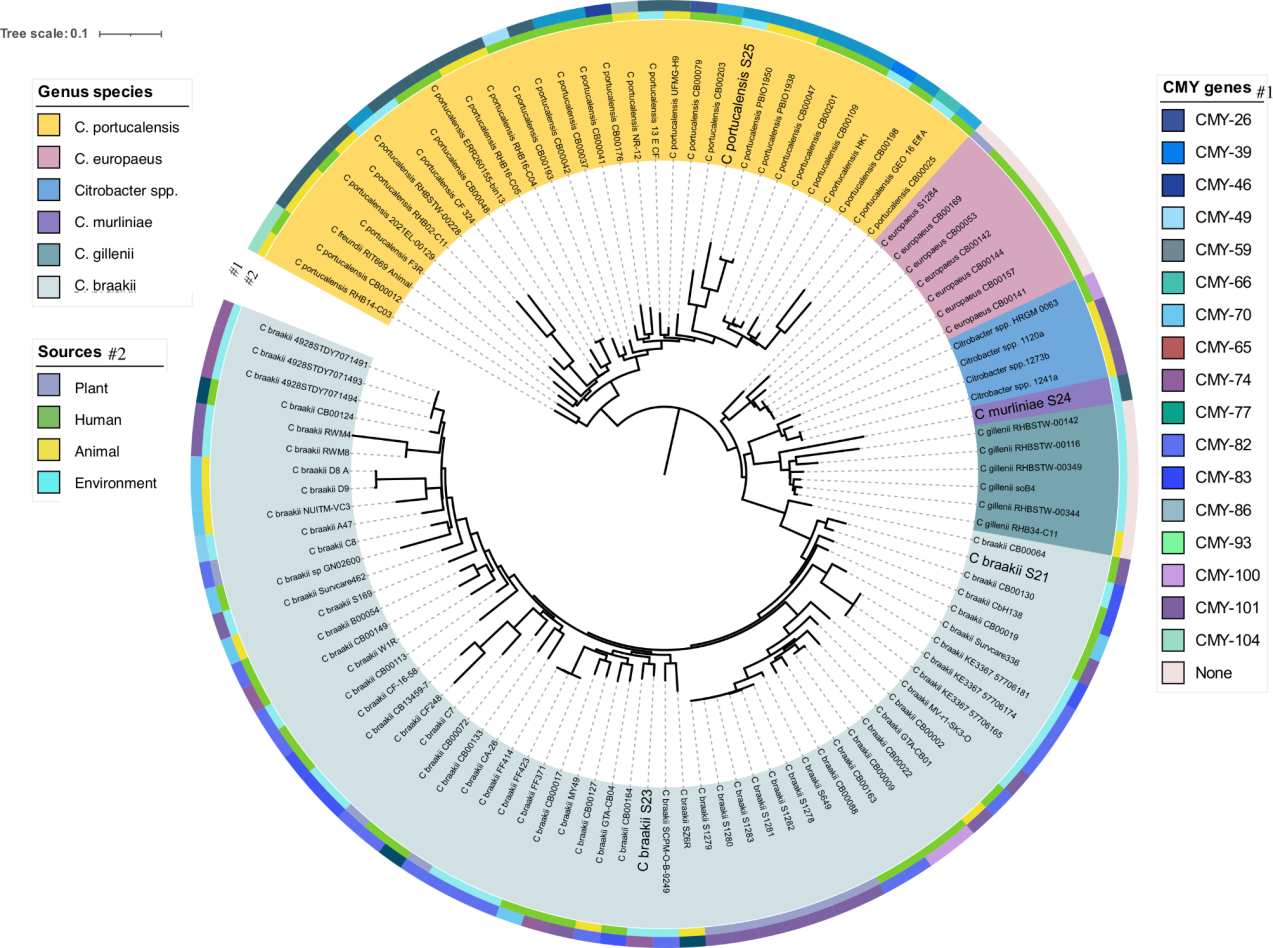
A pangenome analysis of the global 109 *Citrobacter* species strains, including the four *Citrobacter* isolates, highlighted by means of high font size on the phylogenetic tree. A maximum likelihood was used to construct the phylogenetic tree (unrooted), as based on the core and accessory genes. *Citrobacter* species are colour coded in terms of their different respective clusters. The first, inner circle represents the sources of the strains, and the second, outer circler the beta-lactamase genes (*bla-*_*CMY*_) identified in this study

By using core and accessory genes, a pan-genome of 109 *Citrobacter* spp. was constructed to assess genetic diversity (Fig. 1). The sequenced and compared global *Citrobacter* species evidenced genetic diversity which facilitated the clustering of the *Citrobacter* species into distinct species. In this study, 58 924 genes were determined across the *Citrobacter* spp. and sequenced *Citrobacter* isolates genomes that were compared. The latter were used to construct a phylogenetic tree. A total of 1 790 core genes of the *Citrobacter* spp. Were identified, whereas the shell and cloud genes respectively totalled 2 976 and 53 243.

The sequenced *C. braakii* strains S23 and S21 clustered together with *C. braakii* genome strains CB00164 and CB00130, respectively, as isolated from humans in the United States. The present study found that gene clusters distinguished *C. braakii* from the other *Citrobacter* species. Examples of these genes include chitoporin, sapF, *yiaB*, aminopeptidase *ypdF*, urease accessory protein *UreD*, putative protein *YmdA*, putative nucleoside permease nupX, streptomycin-3’-adenyl transferase, *cfaA* fimbrial subunit E and putative Type II secretion system proteins E and G, among others. The sequenced *C. murliniae* strain S24 grouped closely with the *C. gillenii* genomes that had mostly been isolated from wastewater treatment plants in the United Kingdom.

These two species, along with the unassigned *Citrobacter* species, lack most of the core genes when compared to the other assigned *Citrobacter* species. The core genes that are absent in the latter group include *idcA*, murein tetrapeptide carboxypeptidase, *ppaC*, putative manganese-dependent inorganic pyrophosphate, *sutR*, HTH-type transcriptional regulator, *bioC*, malonyl-acyl;-carrier, protein-O-methyltransferase, *pdxK*, pyridoxamine and protein FdrA. In the present study, strain S25 grouped with *C. portucalensis* strain CB00203 as isolated from a human rectal swab in the United States as well as from animal sample strains PBIO1950 and PBIO1938 as isolated from a fly (*Musca domestica*) in Rwanda.

### Population structure of ESBL-producing *Citrobacter* species

Through a de novo assembly-based pangenome study, the evolutionary relationships among 109 ESBL-*Citrobacter* genomes were deduced. Except for the *Citrobacter* spp. cluster, which could not be assigned with species identification, strains consistently grouped in accordance with their MLST profiles. MLST and pangenome analysis allocated the following five species to their respective clusters: *C. braakii* (n = 59), *C. gillenii* (n = 6), *C. europaeus* (n = 7), *C. murliniae* (n = 1), *C. portucalensis* (n = 31) and *Citrobacter* spp. (n = 4). The pangenome phylogenetic tree is represented by samples from animals (n = 21), humans (n = 44), the environment (n = 35) and plants (n = 11). Each *Citrobacter* species cluster consisted of human and animal isolates, except for the clusters of *C. murliniae* and *C. gillenii*.

The vast majority of *Citrobacter* species (n = 91; 83%) were classified as ESBL-producing isolates comprising one of the 18 identified *bla-*_*CMY*_- genes: that is, *bla-*_*CMY*_-, *bla-*_CMY−*26*_, *bla-*_*CMY−39*_, *bla-*_*CMY−46*_, *bla-*_*CMY−59*_, *bla-*_*CMY−66*_, *bla-*_*CMY−70*_, *bla-*_*CMY−65*_, *bla-*_*CMY−71*_, *bla-*_*CMY−74*_, *bla-*_*CMY−77*_, *bla-*_*CMY−82*_, *bla-*_*CMY−83*_, *bla-*_*CMY−86*_, *bla-*_*CMY−93*_, *bla-*_*CMY−100*_, *bla-*_*CMY−101*_ and *bla-*_*CMY−104*_. No CMY genes were present in the *C. europaeus* and *C. gillenii* clustered strains. The *bla-*_*CMY−101*_ (n = 19; 32,2%) and *bla-*_*CMY−82*_ (n = 18; 30,5%) were prevalent in the genomes of *C. braakii*, whereas *bla-*_CMY−59_ (n = 12; 38,7%) and *bla-*_*CMY−77*_ (n = 10; 32,3%) occurred in the genomes of *C. portucalensis*.

### Population structure of the quinolone-resistant *Citrobacter* species

**Fig. 2:**
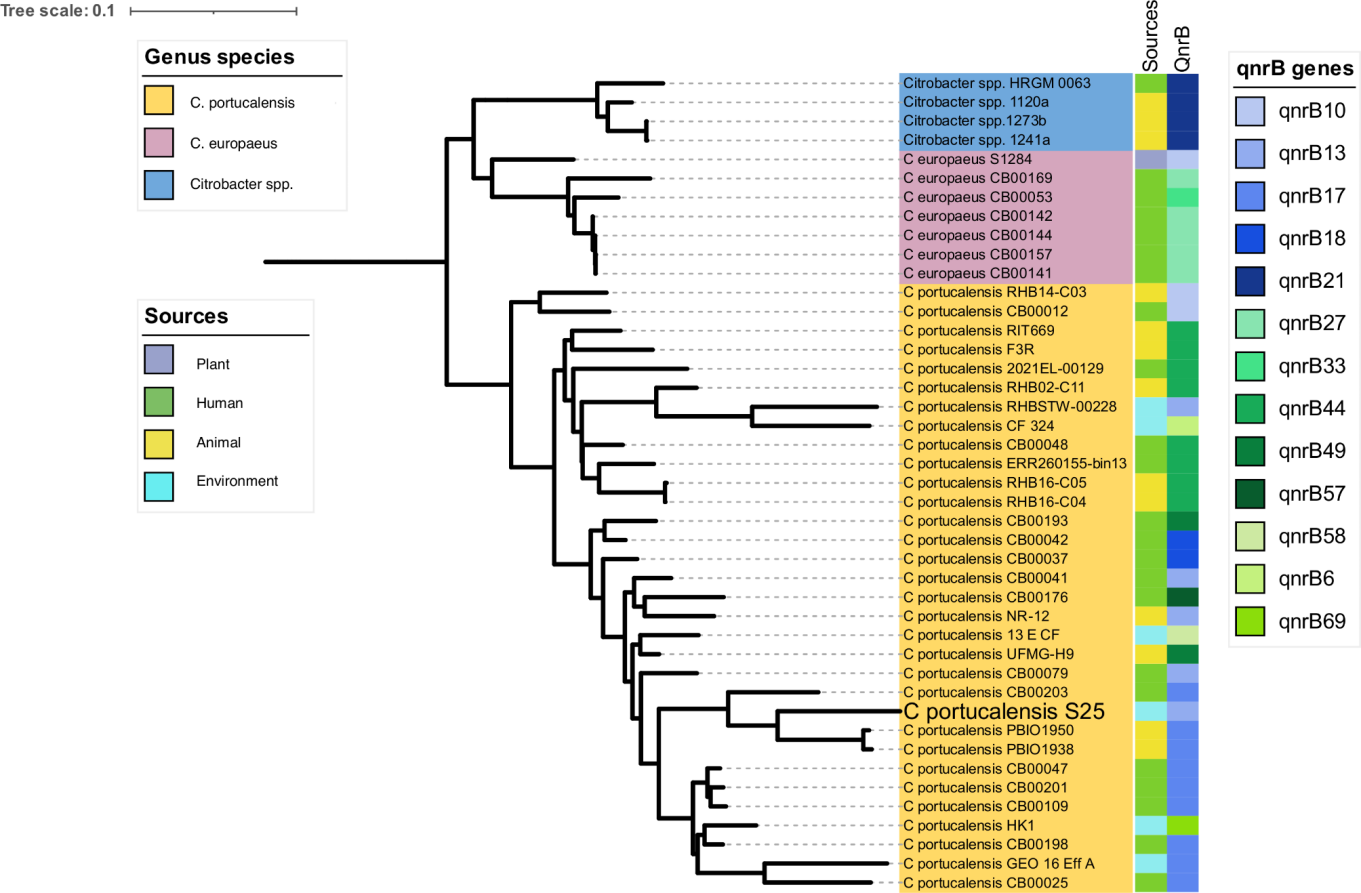
Pangenome phylogenetic tree showing clustering of the quinolone resistance *Citrobacter* species strains (n = 42) as based on a maximum-likelihood phylogenetic tree. Approximately 13 *qnrB* genes were determined and clustering was based on MLST genome identification of the *Citrobacter* species

The quinolone resistance genes (*qnrB*) were found in 42 of the 109 genomes of the *Citrobacter* species examined (Fig. 2). Gene coding for flouroquinolone resistance were found in only three genomes of *Citrobacter* spp.: *C. europaeus* and *C. portucalensis* showed thirteen quinolone resistance genes that were significant, namely *qnrB10*, *qnrB13*, *qnrB17*, *qnrB18*, *qnrB21*, *qnrB27*, *qnrB33*, *qnrB44*, *qnrB49*, *qnrB57*, *qnrB58, qnrB6* and *qnrB69*. Each of the unnamed strains of *Citrobacter* species had the *qnrB21* profile. The *qnr27* gene was present in 83,3% (n = 5) of the *C. europaeus* strains that had been isolated from humans (n = 6), except for strain CB00169. The *qnrB13* gene was present in the genome of the sequenced *C. portucalensis* strain S25 as isolated from the environment: however, this gene was also detected in human and animal samples. This strain clusters with the *C. portucalensis* genomes CB00079, PBIO1950 and PBIO1938 strains, which are *qnrB17*-positive. *C. portucalensis* appears to contain a variety of *qnrB* genes: eleven of these were determined in this cluster.

### Antibiotic resistance genes on the four *Citrobacter* genomes

**Fig. 3:**
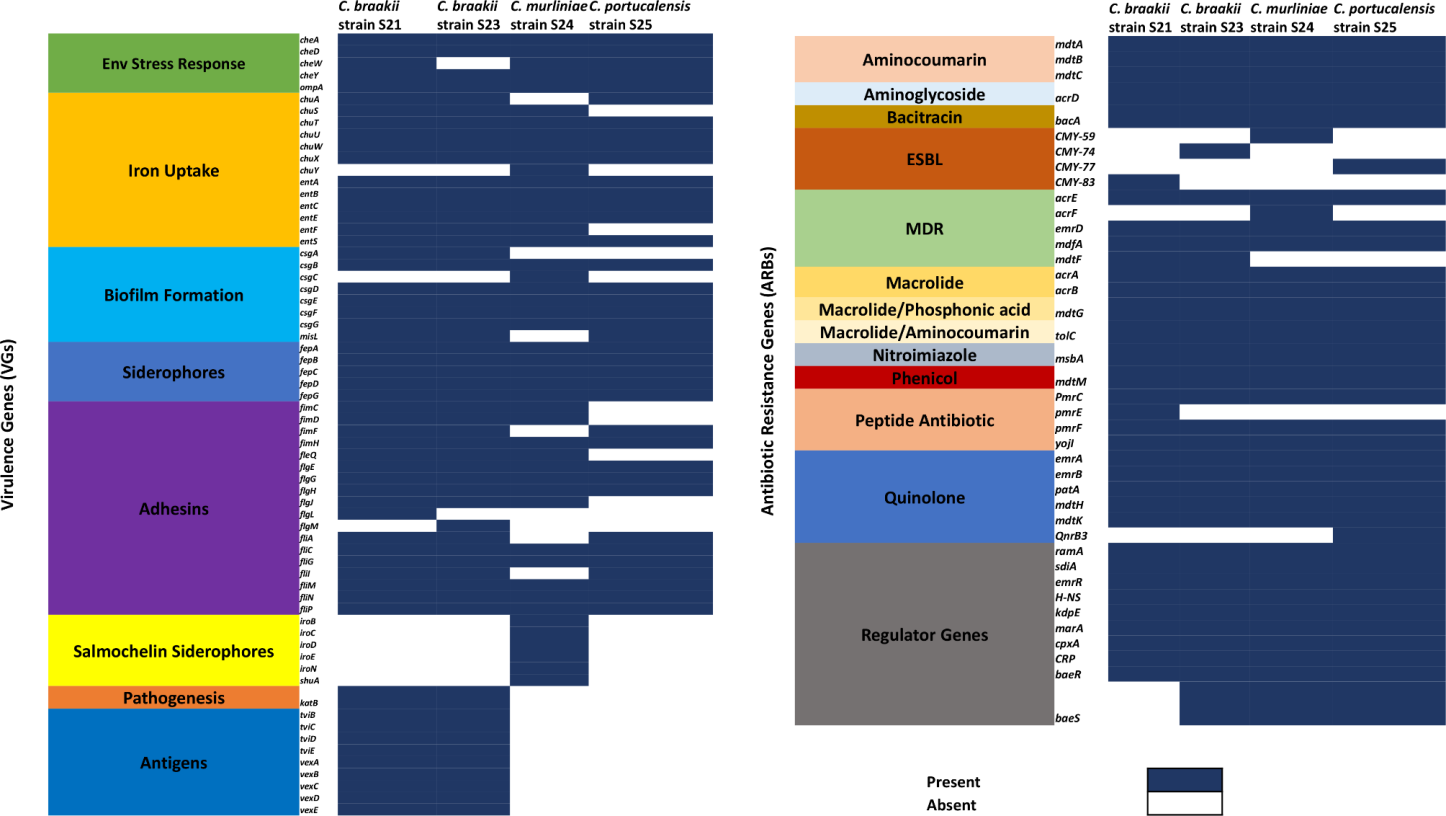
Heatmap reflecting the presence and absence of virulence genes (65 in total) and antibiotic resistant genes (40 in total) across the four sequenced *Citrobacter* spp. isolates

The sequences of the *Citrobacter* species were analysed for antibiotic resistance genes on the four sequenced *Citrobacter* genomes. Forty antibiotic resistance genes were identified by the Comprehensive Antibiotic Resistance Database (CARD) across the four *Citrobacter* species (Fig. 3). The *acrAB-TolC* complex which encode for the intermembrane tripartite multidrug for macrolides, including transcriptional activator *marA* were the common identified ARGs determined in the four sequenced *Citrobacter* genomes. This genotypic profile of each of the four isolates is well supported by the phenotypic profile (Table 1) observed by using disc diffusion assay (DDA). The *Citrobacter* spp. S21, S23, S24 and S25 strains appear to have distinctive beta-lactamase genes such as *bla-*_*CMY−83*_, *bla-*_*CMY−74*_, *bla-*_*CMY−59*_ and *bla-*_*CMY−77*_, respectively (Fig. 3).

The *acrD* efflux pump gene and the regulon *baeR* that were identified in these genomes corroborated the finding of phenotypic resistance to aminoglycosides, as was demonstrated by all four *Citrobacter* spp. The *C. portucalensis* strain S25 consisted of the quinolone-resistant gene *qnrB13*; which is confirmed by phenotypic susceptibility to nalidixic acid. The quinolone resistance genes such as *emrAB/TolC*, *patA*, and *mdtHK* efflux complexes were detected in the sequenced *Citrobacter* genomes. Most of these variables seemed to be connected to multidrug efflux pump processes, which included genes such as *mdtABCGHK*. The heatmap depicts the fact that *C. braakii* S21 and S23 strains were similar in their ARG profile to a greater degree than the others (Fig. 3). This was confirmed by the presence of multidrug resistance *mdtF* gene in these two genomes.

The phenotypic features that were identified by using DDA affirmed the presence of the extended beta-lactamase (*bla-*_*CMY*_) genes in all four sequenced genomes. The *pmrE* gene, which affects the peptide antibiotics class by means of antibiotic target alteration, was the only AR gene found in *C. braakii* strain S21 (Fig. 3). The inner membrane transporter gene, *acrF*, which is known as a multi drug resistant gene, was only found in the *C. murliniae* S24 strain. However, this gene is related to the *acrB* gene, which was also identified in the four *Citrobacter* genomes in this study. A quinolone resistance gene *qnrB13* was present in the genome of *C. portucalensis* strain S25. Pangenomic analysis of the *C. portucalensis* strains showed that the quinolone gene (*qnrB*) differentiates this species from the other three genomes strains that were compared in this study.

### Determination of the virulence assays and genes on the four *Citrobacter* genomes

All four isolates showed a similar extracellular enzyme production profile, except for isolate S24, which did not produce catalase and gelatinase. Overall, though, lecithinase, gelatinase, lipase, DNase, proteinase and catalase were produced. A heatmap was generated of the virulence gene profile across the four sequenced genomes so as to identify the main pathogenicity genes in *C. braakii*, *C. murliniae*, and *C. portucalensis* (Fig. 3). The present project discovered primary virulence factors linked to adhesion, biofilm formation, iron intake, the movement of siderophores, environmental stress response and cell surface localisation in the associated strains (Fig. 3). It was found that gene-encoding cell surface localisation of the CPS (*vexABCDE*), and genes involved in capsule polysaccharide biosynthesis (*tviBCDE*) could be exclusively used to differentiate the *C. braakii* genomes from those of *C. murliniae* and *C. portucalensis*. The *C. murliniae* S24 strain exhibited a virulence profile that was distinct from the other genomes that had been compared. This genome consisted of the *iroABCDEN* gene cluster that encodes a heme receptor protein *shuA* for the iron-salmochelin siderophore system. Moreover, the iron uptake gene, *chuY*, was exclusive to this strain.

### Mobile genetic elements and plasmids

Table 3 reflects the fact that all the *Citrobacter* genomes of this study, excluding *C. braakii* S23, indicated the presence of mobile genetic elements such as insertion sequences (ISs) and transposons (Tns). The three genomes that had insertion sequences shared a commonly identified element, IS5075. Mobile genetic elements such as ISEsa1 and ISEsa2 were shared among *C. murliniae* and *C. portucalensis* genomes. The unique profile of IS elements in *C. portucalensis* strain S25 were found in IS903, ISSpu2 and ISSKox1. In contrast, ISEc33, ISKpn24 and ISKpn26 were present in *C. murliniae* strain S24. Except for the *C. braakii* strain S23 genome, only the transposase Tn5403 was found in the sequenced *Citrobacter* genomes in this study. Type IV coupling proteins (T4CPs) were present in all the sequenced *Citrobacter* spp. genomes, except in *C. braakii* strain S21.

**Table 3:** Genome features on the four sequenced *Citrobacter* species

Bacterial genome strain	Assembly size (bp)	C	G + C content	N50	CDS	ANI (%)	PubMLST species ID	ARGs No.	VGs No.	Insertion Sequence	Trans-poson	T4CP gene cluster	T4SS secretion system	Plasmid ID and [Type]
**S21**	4 987 947	144	52.12	60,775	4923	*C. braakii* (98.6) *	*C. braakii*	25	56	*IS5* *IS102* *IS5075*	Tn5403	-	*VirB1, VirB2*, *VirB3, VirB4*, *VirB5, VirB6*, *VirB8, VirB9*, *VirB10, VirB11*, *VirD4*	AA885 [rep_1804]
**S23**	4 805 424	62	52.17	145,585	4602	*C. braakii* (98.7) *	*C. braakii*	24	55	*-*	-	Present	*-*	-
**S24**	5 481 339	104	50.62	192,439	5492	*C. murliniae* (99.26) **	*C. murliniae*	24	47	*ISEsa1* *ISEsa2* *ISKpn24* *ISKpn26* *IS5075* *ISEc33*	Tn5403	Present	*VirD4-TraABCD* *TraEFGH-TrbABCD* *TrbEFGH* *TrbIJKLV%* *TraX_F-TrbBCDE* *TrbFGHIJLN%%*	AA543 [IncHI1A, IncP] AA810 [rep_cluster_1368] AC978 [-] AC431 [-]
**S25**	5 269 614	91	51.49	154,085	5352	*C. portucalensis* (98.6) ***	*C. portucalensis*	24	38	*ISSpu2* *ISEsa1* *ISEsa2* *IS903* *IS5075* *ISKox1*	Tn5403	Present	*TraABCEFG* *TraHIKLNVW#*	AB130 [Col(VCM04)] AA423 [-] AF384 [-] AF578 [-]

Definitions: *Citrobacter braakii strain DY2019 was used as a reference; ** *Citrobacter murliniae* strain P080C CL was used as a reference;*** *Citrobacter portucalensis* A60T was used as a reference; ARG: antibiotic resistance genes; VGs: virulence genes; IS: Insertion sequence (isfinder); Tn: transposons (tn_registry), (-) indicates absent; % Type IV secretion system gene cluster determined in plasmid AA543; %% Type IV secretion system gene cluster determined in plasmid AC978; # Type IV secretion system gene cluster determined in plasmids AB130; No. is the number of genes; CDS: coding domain sequences; C: contigs

Genomes of *Citrobacter* spp. consisted of a diverse category of translocation systems known as the bacterial Type IV secretion systems (T4SSs), except in the genome of *C. braakii* strain S23. Genome *C. murliniae* strain S24 presented a large number of T4SSs, which merits further investigation. The TraD, TraF, TraH and TraG Type IV secretion were found to be common among *C. murliniae* S23 and *C. portucalensis* strain S25. Except for *C. braakii* S23, all the other sequenced *Citrobacter* species carried plasmids, while *C. murliniae* consisted of different plasmids (Table 3 and Supplementary Table 1). Plasmid replicons and incompatibility types were detected on the draft of assembled plasmids. Four draft plasmids were identified in *the C. murliniae* strain, which is associated with the T4SS clusters (Vir-Tra-Trb). The plasmid AA543 of *C. murliniae* strain S25 had a VirD4-TraABCDEFGH-TrbABCDEFGHIJKLV gene cluster, while plasmid AC978 had a TraX_F-TrbBCDEFGHIJLN gene cluster. *Citrobacter portucalensis* strain S25 carried four plasmids, and plasmid AB130 consisted of the TraABCEFGHIKLNVW gene cluster.

Typing analysis of this plasmid AC978 demonstrated that it was related to the *C. freundii* (CP037735) (Supplementary Table 1). Plasmids AA543 and AC978 of *C. murliniae* strain S24 were classified as conjugative plasmids and were closely related to *Klebsiella oxytoca* strain CAV1374 plasmid pKPC (CP011635) and *Escherichia coli* JJ1886 (CP006788), respectively. None of the plasmids discovered in the reference strain P080C_CL and sequenced *C. murliniae* strain S24 were comparable. Sequence typing of the *C. portucalensis* strain S25 plasmid AB130 and AF578 were identified to be closely related to *C. freundii* (CP037735) and *Salmonella enterica* subsp. *enterica serovar* Cubana str. CFSAN002050 (NC_021819), respectively. Meanwhile, plasmids AF384 and AF578 of this strain were found to be closely related to *Enterobacter roggenkampii* (CP019840) and *Pelobacter propionicus* DSM 2379 (CP000484), respectively.

## Discussion

Four *Citrobacter* species that carried ESBL genes were identified during the present study, which assessed the characteristics of *Enterobacteriaceae* bacteria that circulate in the Mooi River system in the North West Province, South Africa. The prevalence of ESBL-producing *Enterobacteriaceae* is receiving increasing attention as a threat to global health [40]. Environmental surface water is frequently disregarded in these studies, though. In the present study, the genomes of the four *Citrobacter* species were first assumed to be *E. coli* on the basis of conventional phenotypic microbiological tests. However, by utilising high-resolution taxonomic categorisation that included ANI and pangenome analysis, these isolates were verified as belonging to the *Citrobacter* species. The use of traditional methods for *Citrobacter* species identification is not recommended, because they are frequently flawed [3, 17].

The present project dispersed the environmental strains of the *Citrobacter* species among its various genetic clusters, which raises concerns about the spread of antibiotic-resistance genes in humans and animals as well as the acquisition of such genes. Additionally, AR genes, virulence genes and mobile genetic elements were investigated. In the case of river systems especially, the population genetic structure of *Citrobacter* genomes has not been fully documented. The first *Citrobacter* species genomes to be isolated from surface water in South Africa exhibit heterologous unique structures that are closely related to the human and animal-associated *Citrobacter* spp. genomes. These *Citrobacter* species were successfully identified in the present study as *C. braakii* (n = 2), *C*. *murliniae* (n = 1) and *C. portucalensis* (n = 1). Thus far, only one genome was sequenced and documented from a human feaces sample in Norwich, United Kingdom [41] for *C. murliniae*: it is the only current genome that is available in GenBank. *C. portucalensis* has been well characterised by means of various biochemical tests [11] However, a test differentiation among *Citrobact*er species such as *C. braakii*, *C*. *freundii*, and *C. europaeus* is lacking.

Antibiotic resistance remains a public health concern [42]. Some of the antibiotics that screened for antibiotic susceptibility in the four *Citrobacter* species belong to the critically essential antibiotic classes [43]. Resistance against fluoroquinolones, aminoglycosides, β-lactams, and macrolides is problematic, particularly when it comes to treating *Enterobacteriaceae* infections [40]. The sequenced *Citrobacter* spp. genomes of the present study displayed resistance against these critically and highly important antibiotic classes, the latter as described by the WHO [42]. The phenotypic presentation of the classes of antibiotics referred to in the present study included aminoglycosides, β-lactams and macrolides.

A comprehensive antimicrobial profile of the four *Citrobacter* genomes was explored by means of WGS. We found that all species had genes that encoded diverse types of antibiotic efflux pumps, including resistance-nodulation-cell division (RND) types, major facilitator superfamily (MFS) types, ATP-binding cassette (ABC) types, all of which confer resistance to aminoglycoside antibiotics, fluoroquinolone and macrolides (Fig. 3). If resistant bacteria enter a freshwater stream, their presence could be associated with the potential risk of transporting resistant genes from harmless bacteria to pathogenic ones that end up in humans who interact with aquatic environments [44].

Over the last decade, *Citrobacter* spp. have also been reported to be resistant to the most generally used antibiotics, including ampicillin, cefotaxime, aminoglycoside and tetracyclines [45]. The present study established that the four sequenced *Citrobacte*r spp. and global strains harboured genes that encode resistance to a critically important antimicrobial such as beta-lactamase. The phylogenomic landscape of the ESBL-producing *Citrobacter* spp. was employed to infer the prevalence of CMY genes. The vast majority of *Citrobacter* spp. (n = 91; 83%) were classified as ESBL-producing isolates consisting of one of the 18 identified *bla-*_*CMY*−_ genes, including *bla-*_*CMY−26*_, *bla-*_*CMY−39*_, *bla-*_*CMY−46*_, *bla-*_*CMY−59*_, *bla-*_*CMY−66*_, *bla-*_*CMY−70*_, *bla-*_*CMY−65*_, *bla-*_*CMY−71*_, *bla-*_*CMY−74*_, *bla-*_*CMY−77*_, *bla-*_*CMY−82*_, *bla-*_*CMY−83*_, *bla-*_*CMY−86*_, *bla-*_*CMY−93*_, *bla-*_*CMY−100*_, *bla-*_*CMY−101*_ and *bla-*_*CMY−104*_. Most ESBL-producing isolates have been reported in *Enterobacteriaceae* [36, 40]. In the present case, the *bla-*_CMY−101_ (n = 19, 32,2%) and *bla-*_CMY−82_ (n = 18; 30,5%) were prevalent in the *C. braakii* genomes. The *bla-*_CMY−101_ and *bla-*_CMY−82_ genes have been reported from *C. braakii* strains that had been isolated from a river in China [46].

The present study suggests that the beta-lactamase genes *bla-*_CMY−101_ and *bla-*_CMY−82_, which contain isolates, are circulating in the river system, However, they are not limited to this environment, as some of *bla-*_CMY−101_ and *bla-*_CMY−82_ were isolated from plants and humans (Fig. 1). The widespread use of broad-spectrum antibiotics might be the key cause of the emergence and dissemination of elevated levels of antimicrobial-resistant strains in the freshwater of the Mooi River [28]. The present study depicts the spectrum of *Citrobacter* in the freshwater of the Mooi River and found that *Citrobacter* is most often associated with multidrug resistance. This resistance might be due to the acquisition of resistance genes such as β-lactamase, efflux pumps or alternation in porins that act synergistically as channels for drug entry so as to confer resistance among *Citrobacter* isolates [4].

A fluoroquinolone resistance indicator, *qnrB13*, was found in *C*. *portucalensis* strain S25 and was compared with other *C*. *portucalensis* genomes. The chromosomal antibiotic resistance *qnrB* gene has also been reported in other *Citrobacter* species [12, 47], which suggests that *Citrobacter* spp. may be the origin of chromosomal antibiotic resistance. The hypothesis was based on species distribution (> 60% in *Citrobacter* spp.) [47–49] and the fact that the *qnrB* gene is prevalent in *C. freundii* strains isolated from human clinical specimens [50]. Other studies support the notion that the genus *Citrobacter* is the origin of *qnrB*, as this gene is distributed in species such as *C*. *freundii*, *C. braakii*, *C*. *youngae*, and *C*. *werkmanii*, However, allele variation specific to each species does exist [12, 49].

The present study demonstrated that the *C. braakii* genomes can be distinguished from *C*. *murliniae* and *C. portucalensis* on the basis of gene encoding for cell surface localisation of the CPS (*vexC*) as well as genes involved in capsule polymer synthesis (*tviB* and *tviE*). Extant studies also report these genes in this kind of context [4]. The present study found that *C. murliniae* strain S24 could not produce catalase, which was confirmed by the absence of the *katB* gene. A gene cluster of the salmochelin siderophore system (*iroBCDEN*) was found in *C. murliniae* S24: it is important for the pathogenicity pathway that confers an advantage in colonisation. This gene cluster was first described in *Salmonella enterica* [51], and orthologous genes were also reported in some *E. coli* strains, which suggests acquisition through horizontal gene transfer [52]. The present study is the first report that *iron*-BCDEN gene clusters were identified in *Citrobacter* species, particularly in the *C. murliniae* genome.

Other virulence factors identified in all *Citrobacter* spp. strains were associated with iron uptake (*chu* and *ent*). Pathogenic bacteria need iron, which they must acquire during infection, if they are to thrive in a mammalian host [53]. However, *C*. *murliniae* contained an additional iron update gene, namely *chuF*. This gene is an anearobilin reductase that exhibits kinetic cooperativity during the process of biochemical degradation in *E. coli* O157:H7 [54]. Among members of the genus *Citrobacter*., especially *C*. *koseri*, extant studies have found strains that consisted of this iron gene cluster [4]. However, in the present study, this gene cluster was found in the four sequenced genomes.

The identified plasmids, as determined in the present study, were mostly associated with T4SSs. The latter is a broad distribution group that secretes macromolecules in Gram-negative and positive bacteria as well as other eukaryotic cells [55]. They play a crucial role in the interactions between bacteria and their surrounding environments by secreting many virulence factors, particularly in the case of pathogenic bacteria [56]. The Type IV secretory genes are not fully exploited: however, they have been reported in most genomes of the *Citrobacter* species [4]. The present study established that bacterial T4SSs were present among the sequenced *Citrobacter* genomes strains, except in the case of *C. braakii* strain S23. This was most probably due to the fact that the plasmid was absent from the strain.

We detected an incompatibility group of plasmids replicon genes, including IncP and IncH. The IncP plasmid replicon type plays important roles around the transfer of antibiotic resistance genes and other types of genetic information among bacteria [57]. They also contain genes that produce virulence factors, engendering bacteria that carry IncP plasmids that are more dangerous to humans than other organisms. The IncP plasmids are sometimes referred to as wild-type ones due to their ability to transfer themselves among many different bacterial species [57]. The colicinogenic (or *Col*) factors, such as plasmid Col(VCM04), determine the production of proteins called colicins, which can kill other bacteria. Given certain environmental conditions, plasmids can provide many different types of selective advantages, including antibiotic resistance, resistance to pollutants or UV and biofilm formation [58].

The sequenced *C. Murliniae* strain S24 consisted of three distinct types of T4SS gene clusters (*Vir*, *Tra* and *Trb*) that were encoded in the plasmid AA543: *Vir*D4, *Tra*ABCDEFGH, and *Trb*ABCDEFGHIJKLV. Meanwhile, the plasmid AC978 encoded the *Tra*XF and *Trb* gene clusters *Tra*XF and *Trb*BCDEFGHIJLN. It is well known that some T4SS components, such as the tra-operon involved in conjugation, exhibit significant sequence and structural homology and have identical protein activities that are associated with pathogenicity [59, 60]. Therefore, the T4SS is not limited to certain bacterial species, as it has been reported in other pathogenic species, such as *Pseudomonas aeruginosa*, *Vibrio cholerae*, enteroaggregative *Escherichia coli*, *Burkholderia thailandensis*, *Serratia marcescens*, *Burkholderia malle*, and *Salmonella enterica*, which are also involved in conjugative transfer and plasmid replication [61].

## Conclusion

Our findings suggest that *C. portucalensis*, *C. murliniae*, and *C. braakii* are multi-drug resistant pathogens with intrinsic genes that encode forESBL. The use of whole-genome sequencing identified these species and the phylogenomic structure of the four *Citrobacter* species that circulate in surface water in the Mooi River of the North West Province, South Africa. The *C. murliniae* strain showed a unique profile with an *iron*-BCDEN gene cluster of salmochelin siderophore system. Fluoroquinolone resistance (*qnrB13*) was only detected in *C. portucalensis*, which was closely related to animal and human *C. portucalensis*.

These genomes represent important reference points. They act as crucial pieces of detailed genetic information and phenotypic data that will be used as models for the characterisation of antibiotic resistance mechanisms and interactions. Of further importance is that the study, as centred on ESBL-producing *Citrobacter* species, is that all four genomes carried the *bla-*_*CMY*_-gene, thus presenting a threat in its transmission potential for the environment and possibly other sectors, the latter as described in the One Health model.

## Materials and methods

### Sample collection and isolation

Samples were collected from a Mooi River site (26° 42’ 29.3” S 027° 06’ 20.6” E), as described in earlier part of the present project [62]. However, in the original study, the aim was to characterise *Enterobacteriaceae* and their potential antibiotic resistance within surface water in the Mooi River system. In summary of that study, water samples (100 ml) were processed by the Colilert-18 (IDEXX, USA) procedure. Selective media of Sorbitol MacConkey agar (Oxoid, UK) infused with antibiotics (0.05 mg/L Cefixime trihydrate (Sigma Aldrich, India) and 2.5 mg/L Potassium Tellurite (Sigma Aldrich, Japan) (SMAC-CT agar)) were further used to isolate presumptive pathogenic *Enterobacteriaceae* species incubated at 37 °C for 24 h.

The isolates were further subjected to Rainbow agar O157 (Biolog, USA) infused with Novobiocin sodium salt (Sigma Aldrich), and were subsequently incubated at the same conditions as those of the SMAC-CT agar. The reference strains that were used in this study were non-pathogenic *E. coli* ATCC 10,536, pathogenic *E. coli* O157:H7 ATCC 700,728 and ESBL-producing *E. coli* ATCC 35,218 (Microbiologics, USA). ESBL-producing *Citrobacter* isolates were confirmed phenotypically on CHROMagar™ ESBL (CHROMagar, France). The agar differentiates between ESBL *E. coli* (red colony presentation) and ESBL *Klebsiella*, *Enterobacter* and *Citrobacter* spp. (metallic blue, with or without a red halo). The selection of these four isolates was based on their ability to produce the ESBL enzymes.

These ESBL-producing *Enterobacteriaceae* were analysed according to the manufacturer’s instructions on the Enteropluri-Test strip (Liofilchem, Italy). A set of 13 biochemical reactions was detected, which included glucose fermentation, gas production, hydrogen sulphide production, lysine decarboxylation, ornithine decarboxylation, adonitol fermentation, lactose fermentation, arabinose fermentation, sorbitol fermentation, dulcitol fermentation, phenylalanine deamination, urea hydrolysis and citrate utilisation [63]. Furthermore, seven in-vitro virulence assays were conducted, which included catalase, proteinase, DNase, lipase, gelatinase and lecithinase [63].

### Antimicrobial susceptivity testing

Pure cultures of *Citrobacter* isolates (n = 4) were sub-cultured onto Mueller-Hinton agar (Biolabs, USA) by using the Kirby-Bauer disc diffusion method [64] for a set of 12 antibiotics. The antibiotics included ampicillin (10 µg), amoxicillin (10 µg), cefazolin (30 µg), erythromycin (15 µg), gentamycin (10 µg), kanamycin (30 µg), streptomycin (10 µg), tetracycline (30 µg), ciprofloxacin (5 µg), chloramphenicol (30 µg), neomycin (30 µg) and nalidixic (30 µg) (Oxoid, UK). The results were interpreted in accordance with the M100 CLSI guideline for Antimicrobial Susceptibility Testing (2017) (https://clsi.org/standards/products/free-resources/access-our-free-resources/) and the clinical breakpoint for bacteria (v12.0) EUCAST guidelines (https://www.eucast.org/clinical_breakpoints/).

### Genomic DNA extraction and whole-genome sequencing

Overnight broth cultures of the bacterial isolates were centrifuged and pelleted for DNA extraction. The Chemagic kit (Perkin Elmer, Germany) was used to extract the total genomic DNA of the *Citrobacter* isolates while following the manufacturer’s protocol. The concentration and quality of the DNA were determined by using the Qubit 2.0 fluorometer (Thermofisher-Scientific, USA). The DNA integrity was monitored on a 2% agarose gel pre-stained with ethidium bromide and visualised on a UV transilluminator. Paired end libraries were generated by using the NEBNext kit (BioLabs, England) according to the NEBNext for Illumina protocol. Cluster generation and sequencing were performed by means of the MiSeq Reagent Kit V3 (2 × 300 bp) on the MiSeq 2000 (Illumina, USA).

### Genome assembly and annotation

Quality of the sequenced reads were assessed by using FastQC software 0:10.1 [65]. Trimmomatic [66] was used to remove the ambiguous nucleotide reads in terms of a threshold of reads > Q28. The paired end trimmed reads of *Citrobacter* spp. strains were assembled *de novo* by using the SPAdes v1.1.0 pipeline [67]. The minimum contig length was set to 500 bp, and kmer sizes 21, 33, 55, 77, 99 and 127 were used for the assembly. CheckM [68] was additionally used to assess potential contaminants in individual assembled genomes. Quast v 2.3 [69] was used to evaluate the draft genome assemblies of *Citrobacter* species. The assembled contigs were annotated by means of the NCBI prokaryotic genome automatic annotation pipeline (PGAAP) [70].

### Whole genome *in-silico* taxonomic analysis and pangenomics

The genomic sequence and the pubMLST database of *Citrobacter* spp. on the website https://pubmlst.org/ were used to determine the species identity. FastANI version 1.3 [71] was used to estimate the isolate’s ANI against closely related genomes of *Citrobacter* species as references. Based on the ANI, a percentage identity of > 90% against the reference genome was considered to belong to the same species. For pangenome analysis, 109 *Citrobacter* publicly available genomes were retrieved from GenBank (Supplementary Table 2), and were selected on the basis of pubMLST match profiles of the sequence genomes. The inclusion was also based on the One Health concept, so as to determine relatedness to animal, human and environmental strains. This included the geographical region of Africa and worldwide genomes representation.

All the retrieved and sequenced *Citrobacte*r strain genomes in this study were further annotated by using Prokka v.1.14.0 [72]. Similarity searches in terms of the coding domain sequences (CDSs) of assembled genomes were conducted by using pair-wise BLASTp [73] and the Markov Cluster Algorithm (MCL). Clusters were created by using paralogs of the genomes and were ordered with a view to the presence/ absence of orthologs [74]. Pangenome clusters were defined as follows: core genes present in all isolates; soft core genes present in at least 95% of isolates; shell genes present between 15 and 95% of isolates; cloud genes in less than 15% of isolates. The phylogenetic tree of the *Citrobacter* genomes was visualised by using ITOL [75].

### Antibiotic resistance, virulence gene detection, mobile genetic elements, and accession numbers

The ABRicate pipeline, which was assessed on 25 January 2023, was employed to identify antibiotic resistance and virulence genes in the four genomes of the isolated *Citrobacter* species. Antimicrobial resistance determinants were identified in each assembled genome by using the ResFinder database (–db ResFinder) [76] with minimum identity and coverage thresholds of 75 (– minid 75) and 50% (–mincov 50), respectively. The Comprehensive Antibiotic Resistance Database (CARD) was also employed to determine the AR genes. ABRicate was further used to detect virulence factors in the sequenced genomes of *Citrobacter* spp. By using the Virulence Factor Database (VFDB; –db vfdb) [77, 78] in terms of minimum identity and coverage thresholds of 70 (–minid 70) and 50% (–mincov 50), respectively.

Mobile genetic elements were investigated on the sequenced genomes of the *Citrobacter* by means of Mobile Element Finder v1.0.3 (2023-01-20) on the Center for Genomic Epidemiology platform [79]. Plasmid replicons were identified by ABRicate on the sequenced genomes by using the PlasmidFinder database [80]. The MOB-Typer tool from MOB-Suite software v1.4.9 [81], and OriTfinder [82] was used to characterise plasmid sequences.

### Electronic supplementary material

Below is the link to the electronic supplementary material.

Supplementary Material 1

Supplementary Material 2

Supplementary Material 3

Supplementary Material 4

Supplementary Material 5

## Data Availability

*Citrobacter* species isolates S21, S23, S24 and S25 genome sequences were deposited in NCBI GenBank under the accession numbers SRR23239838, SRR23239837, SRR23239836, and SRR23239835, respectively.
