# Supplementary material for: The phylogenomic landscape of extended-spectrum β-lactamase producing Citrobacter species isolated from surface water
Source: BMC Genomics. 2023 Dec 7;24:755. doi: 10.1186/s12864-023-09867-4 (PMC10704729; doi:10.1186/s12864-023-09867-4)
Supplement: Supplementary file 1 — Supplementary Material 1 [file 12864_2023_9867_MOESM1_ESM.pdf]

Supplementary Table 1 Characterization of the nine plasmid sequences harboured in the *Citrobacter* spp, according to replicon type, relaxase type, Mate-Pair Formation (MPF) type, predicted mobility, and GenBank nearest neighbour.

Plasmid ID	No. of contigs	Size (bp)	GC content	Replicon types	Relaxase types	MPF type	Predicted mobility	Accession closest ID	Genus and species nearest identification
C. braakii S21:AA885	1	61960	52.3	rep_cluster_1804	MOB_C	MPF_T	Conjugative	CP009851	*Enterobacter cloacae* strain ECNIH4 plasmid pENT-c88
C. murliniae S24:AA543	7	317395	51.5	IncHI1A, IncP	MOB_H MOB_P	MPF_T	Conjugative	CP011635	*Klebsiella oxytoca* strain CAV1374 plasmid pKPC_CAV1374
C. murliniae S24:AC978	1	54358	46.4	rep_cluster_1368	MOB_P	MPF_T	Conjugative	CP006788	*Escherichia coli* strain JJ1886 plasmid pJJ1886_4
C. murliniae S24:AC431	1	17697	57.1	-	-	-	Non-mobilizable	CP026176	*Klebsiella pneumoniae* strain KPNIH50 plasmid pKPN-bb08
C. murliniae S24:AA810	1	14631	50.8	-	-	-	Non-Mobilizable	CP035364	*Escherichia coli* strain BR10-DEC plasmid unnamed4
C. portucalensis S25:AB130	16	231257	46.8	Col(VCM04)	MOB_H	-	Mobilizable	CP037735	*Citrobacter freundii* strain CAV1857 plasmid pCAV1857-208
C. portucalensis S25:AF578	3	65815	51.6	-	MOB_P	-	Mobilizable	NC_021819	*Salmonella enterica subsp. enterica* serovar Cubana str. CFSAN002050 plasmid unnamed2
C. portucalensis S25:AA423	1	18168	59.7	-	-	-	Non-mobilizable	CP019840	*Enterobacter roggenkampii* strain R11 plasmid pASM1
C. portucalensis S25:AF384	1	10174	55.0	-	-	-	Non-mobilizable	CP000484	*Pelobacter propionicus* strain DSM 2379 plasmid pPRO2
