# Supplementary material for: The phylogenomic landscape of extended-spectrum β-lactamase producing Citrobacter species isolated from surface water
Source: BMC Genomics. 2023 Dec 7;24:755. doi: 10.1186/s12864-023-09867-4 (PMC10704729; doi:10.1186/s12864-023-09867-4)
Supplement: Supplementary file 2 — Supplementary Material 2 [file 12864_2023_9867_MOESM2_ESM.pdf]

Supplementary Table 2A The antibiotics resistance genes detected using Comprehensive Antibiotic Resistance Database. The genes and their associated drug class, treatments, and resistance mechanism of action are indicated.

Gene	Drug class	Antibiotic treatment	Resistance mechanism
acrAB/TolC	Multidrug/ macrolides	Global health threat	Efflux pump
acrD	Aminoglycoside	Severe infections of the abdomen, urinary tract, and the heart	Efflux pump
acrEF/TolC	Multidrug	Global health threat	Efflux pump
bacA	Bacitracin	Preventative for skin infections	Efflux pump
CMY-59	β-lactams (ESBLs)	Pulmonary, urinary, skin, and soft tissue infection	Antibiotic inactivation
CMY-74	β-lactams (ESBLs)	Pulmonary, urinary, skin, and soft tissue infection	Antibiotic inactivation
CMY-77	β-lactams (ESBLs)	Pulmonary, urinary, skin, and soft tissue infection	Antibiotic inactivation
CMY-83	β-lactams (ESBLs)	Pulmonary, urinary, skin, and soft tissue infection	Antibiotic inactivation
emrAB/TolC	Quinolones	Community and severe hospital-acquired infections	Efflux pump or mutations
emrD	Multidrug	Global health threat	Efflux pump
mdfA	Multidrug	Global health threat	Efflux pump
mdtABC/TolC	Aminocoumarin	Lyme disease	Efflux pump
*mdtE*F*	Multidrug	Global health threat	Efflux pump
mdtG	Phosphonic acid/Macrolides	Urinary tract infections	Efflux pump

mdtH	Quinolone	Septicemia, urinary, joint, bone, tissue, and pulmonary infections	Efflux pump
mdtK	Quinolone	Septicemia, urinary, joint, bone, tissue, and pulmonary infections	Efflux pump
mdtM	Phenols	Bacterial conjunctivitis (eye infection) and Cholera treatment	Efflux pump
msbA	Nitroimidazole	Parasitic, anaerobic bacterial infections and prevent post-operative infections	Efflux pump
patA	Quinolone	Septicemia, urinary, joint, bone, tissue, and pulmonary infections	Efflux pump
pmrEFC	Peptide antibiotic	Multidrug-resistant infections	Antibiotic target alteration
*qnrB13***	Quinolone	Septicemia, urinary, joint, bone, tissue, and pulmonary infections	Efflux pump
yojI	Peptide antibiotic	Multidrug-resistant infections	Efflux pump

*absent

** only determined in *C. portucalensis* strain S25
