# Supplementary material for: The phylogenomic landscape of extended-spectrum β-lactamase producing Citrobacter species isolated from surface water
Source: BMC Genomics. 2023 Dec 7;24:755. doi: 10.1186/s12864-023-09867-4 (PMC10704729; doi:10.1186/s12864-023-09867-4)
Supplement: Supplementary file 3 — Supplementary Material 3 [file 12864_2023_9867_MOESM3_ESM.pdf]

Supplementary Table 2B The regulatory genes that up or down-regulate for the antibiotic resistance genes.

Regulator Genes	
baeRS	Regulates the expression of MdtABC and AcrD efflux complexes
cpxA	Regulates efflux complex expression.
CRP	CRP is a global regulator that represses MdtEF multidrug efflux pump expression.
emrR	Regulator for the EmrAB-TolC multidrug efflux pump
H-NS	Repressor of the membrane fusion protein genes acrE mdtE and emrK as well as nearby genes of many RND-type
kdpE	Repressor of the membrane fusion protein genes acrE mdtE and emrK as well as nearby genes of many RND-type multidrug exporters.
marA	Regulates MDR efflux pump AcrAB also down-regulates synthesis of the porin OmpF.
ramA	Regulator of AcrAB-TolC and leads to high-level multidrug resistance
sdiA	Regulator of AcrAB only when it's expressed from a plasmid
