# Supplementary material for: The phylogenomic landscape of extended-spectrum β-lactamase producing Citrobacter species isolated from surface water
Source: BMC Genomics. 2023 Dec 7;24:755. doi: 10.1186/s12864-023-09867-4 (PMC10704729; doi:10.1186/s12864-023-09867-4)
Supplement: Supplementary file 4 — Supplementary Material 4 [file 12864_2023_9867_MOESM4_ESM.pdf]

Supplementary Table 3 Virulence genes of *Citrobacter* species encoding adhesins, environmental stress response, iron uptake, siderophores, biofilm formation, and antigens determined in this study.

Virulence Response	Virulence genes	Product and Mechanism
Environmental stress response	*cheA, cheD, cheW, cheY*	Chemotaxis proteins production
Iron uptake	*chuA, chuS, chuT, chuU, chuW, chuX, chuY*	Heme-binding proteins
Biofilm formation	*csgA, csgB, csgC, csgD, csgE, csgF, csgG*	Curli production assembly
Iron uptake	*entA, entB, entC, entE, entF, entS*	Multi-enzyme complex formation carries out a series of biochemical reactions in the bacterial cell.
Siderophores	*fepA, fepB, fepC, fepD, fepG*	Ferrienterobactin transporter/ binding protein
Adhesins	*fimC, fimD, fimF, fimH*	Chaperone and usher proteins associated with virulent adhesion.
Adhesins	*flgG, flgH*	Flagellar protein associated with virulent adhesion.
Adhesins	*fliA, fliC, fliG, fliI, fliM, fliN, fliP*	Flagellar motor switch proteins associated with virulent adhesion.
Salmochelin siderophores	*iroB, iroC, iroD, iroE, iroN, shuA*	Iron-salmochelin utilization plays a role in pathogenicity.
Biofilm formation	*misL*	Autotransporter protein

Environmental stress	*ompA*	Outer membrane protein A
Pathogenesis	*katB*	Resistance against peroxidase and facilitate phagocyte-mediated killing.
Antigens	*tviB, tviC, tviD, tviE*	Polysaccharide biosynthesis protein involved in capsule polymer synthesis.
Antigens/Biofilm Formation	*vexA, vexB, vexC, vexD, vexE*	Polysaccharide export protein produced that encodes for cell surface localization of the Capsular Polysaccharides.
